# Peritoneal loose body presenting as a hepatic mass: A case report and review of the literature

**DOI:** 10.1515/med-2021-0351

**Published:** 2021-09-09

**Authors:** Yang Wen, Min-jie Shang, Yan-qing Ma, Song-hua Fang, Yuan Chen

**Affiliations:** Department of Radiology, Zhejiang Provincial People’s Hospital, Affiliated People’s Hospital of Hangzhou Medical College, Hangzhou, Zhejiang, People’s Republic of China; Department of Hepatobiliary Surgery, Zhejiang Provincial People’s Hospital, Affiliated People’s Hospital of Hangzhou Medical College, Hangzhou, Zhejiang, People’s Republic of China; Department of Pathology, Zhejiang Provincial People’s Hospital, Affiliated People’s Hospital of Hangzhou Medical College, No. 158, Shangtang Road, Hangzhou, 310014, Zhejiang, People’s Republic of China

**Keywords:** laparoscopic, peritoneal loose body, peritoneal cavity, ultrasound, computed tomography, magnetic resonance imaging

## Abstract

Peritoneal loose body (PLB) is a rare clinical entity. It is generally agreed that the most common origin of the loose bodies is appendix epiploica. We here report a case of PLB that looks like a “boiled egg,” which was misdiagnosed preoperatively as a lesion of hepatic origin and was confirmed by operation and postoperative pathology. PLBs are rare entities, a good understanding of their specific imaging features can help prevent misdiagnosis, but sometimes an accurate preoperative diagnosis is still difficult to achieve. Exploratory laparoscopy is a recommended method for management of PLBs.

## 1 Introduction

A peritoneal loose body (PLB) is rare in clinical practices, which often occurs in the peritoneal cavity. Its name originates from its changeable position. In other words, it tends to move obviously with the change in the patient’s position. The incidence of PLB is occult and preoperative diagnosis is difficult. Moreover, it is often found accidentally in imaging physical examination, abdominal exploration operation, or autopsy. Though the reason for the formation of peritoneal free bodies remains unclear, the role of infarction, saponification, and calcification after chronic volvulus of intestinal fat is speculated [1,2,3,4,5,6].

Herein we report an unusual case of PLB and review the literature. We hope that, through our case, a good understanding of their specific imaging features may help prevent misdiagnosis.

## 2 Materials and methods

We performed a narrative review of the literature by searching “peritoneal loose bodies” and “peritoneal mouse” on PubMed and Scopus. Case reports and reviews were chosen and used to extract data regarding gender, age, clinical feature, number of lesions, size of lesions, preoperative diagnosis, surgical procedure, intraoperative lesion location, and free status (record the position seen during the operation, if there is no operation, record the position seen during the examination). Two authors (WY and CY) independently carried out online research. Full texts of relevant articles were further assessed for inclusion in this study, and the characteristics of 50 cases that have been reported are shown in Table 1.

**Table 1:** Reported cases of peritoneal loose bodies in the literature

Author	Published year	Gender	Age (year)	Clinical feature	Single/Multiple	Size (mm)	Preoperation diagnosis	Surgical procedure	Intraoperative location
Shephered [21]	1951	M	79	Acute retention of urine	Single	70 × 55	Vesical calculus	Laparotomy	PC#
Southwood [22]	1956	M	53	Right hypochondrium pain	Single	6.35 × 12.7	Acute appendicitis	Laparotomy	PC#
Burns and Rogers [23]	1969	F	33	Pelvic pain	Single	Not available	Not available	Laparotomy	Left upper quadrant
Bhandarwar et al. [24]	1996	M	65	Acute retention of urine	Single	90 × 80	Vesical calculus	Laparotomy	Rectovesical pouch#
Takada et al. [1]	1998	M	79	Incidental	Multiple	70 × 60	Calcified leiomyoma	Laparotomy	PC#
						70 × 60			
Nomura et al. [25]	2003	M	63	Incidental	Single	50 × 40 × 30	Leiomyoma	Laparoscopy	PC#
Ohgitani et al. [26]	2004	M	65	Incidental	Single	75 (max)	Peritoneal loose body	Laparoscopy	Peritoneal cavity#
Fujita et al. [9]	2005	M	75	Urinary frequency	Single	77 × 70 × 62	Calcified retroperitoneal tumor	Laparotomy	PC*
Asabe et al. [27]	2005	F	2 month	Urinary tract infection	Single	30 (max)	Not available	Laparoscopy	PC#
Ooyagi et al. [28]	2006	M	65	Abdominal discomfort	Single	40 × 35	Peritoneal loose body	Untreated	Not available#
Ghosh et al. [2]	2006	M	63	Intestinal obstruction	Multiple	58 × 45 × 37	Gallstones	Laparotomy	Pelvic cavity#*
						52 × 45 × 37			
Mohri et al. [5]	2007	M	73	Abdominal pain	Single	95 × 75	Peritoneal loose body	Laparotomy	PC#
Takayama et al. [29]	2009	M	63	Abdominal discomfort	Single	45 × 40	Not available	Laparoscopy	PC#
Kao et al. [17]	2010	F	69	Abdominal pain	Single	40 × 30 × 23	Gallstone ileus	Laparotomy	Right lower quadrant*
Koga et al. [12]	2010	F	33	Infertility	Single	30 × 20	NA	Laparoscopy	vesicouterine pouch#
Hedawoo and Wagh [30]	2010	M	65	Abdominal pain	Single	95 × 85	Duplication cyst	Laparotomy	Right iliac region#
							Dermoid cyst		
Sewkani et al. [6]	2011	M	64	Small bowel obstruction	Single	70 × 50	Not available	Laparotomy	PC*
Gayer and Petrovitch [20]	2011	M	59	Postoperative follow-up of malignant tumor	Single	30 (max)	Peritoneal loose body	Untreated	PC# (examination)
Jang et al. [31]	2012	M	60	Dyspepsia	Single	45 × 40 × 30	Stromal tumor, leiomyoma, or teratoma	Laparoscopy	PC#
Nozu and Okumura [32]	2012	M	67	Incidental	Single	40 (max)	Peritoneal loose body	Untreated	PC# (examination)
Kim et al. [33]	2013	M	50	Incidental	Single	75 × 70 × 68	Calcifying fibrous pseudotumor	Laparoscopy	PC#
Rajbhandari et al. [34]	2013	M	67	Incidental	Single	50 × 40	Bowel mass	Laparoscopy	Peritoneal cavity#
Maekawa et al. [35]	2013	M	58	Postoperative follow-up of malignant tumor	Single	20(max)	Not available	Lymphadenectomy	Extraperitoneal space#
Allam et al. [36]	2013	M	77	Abdominal pain	Single	17(max)	Peritoneal loose body	Untreated	PC# (examination)
Sahadev and Nagappa [37]	2014	M	52	Abdominal pain	Single	60 × 50	Calcified leiomyoma	Laparoscopy	PC#
Rubinkiewicz et al. [7]	2014	F	70	Mechanical bowel obstruction	Single	200 × 100	Leiomyoma	Laparotomy	PC
Makineni et al. [38]	2014	M	52	Abdominal discomfort	Single	60 × 50	Calcified leiomyoma	Laparotomy	Recto-vesical pouch#
Srinivasan and Xavier [39]	2015	M	53	Abdominal pain	Single	50 × 40	Not available	Laparotomy	Right iliac region#
Suganuma et al. [13]	2015	F	35	Incidental	Single	75 × 70 × 60	Leiomyoma	Laparoscopy	The pouch of Douglas#
Zhang et al. [11]	2015	M	51	Incidental	Single	50 × 40 × 40	Teratoma and stromal tumor	Laparoscopy	PC#
Cooke and Kirk [14]	2015	F	30	Abdomial pain	Single	30 × 20 × 10	Ectopic pregnancy	Laparoscopy	Vesicouterine pouch#
Sussman and Murdock [8]	2015	M	62	Urinary frequency	Single	100 × 95 × 75	Not available	Laparoscopy	PC#
Rosic et al. [18]	2016	M	73	Retention of urine	Single	66 × 56 × 40	Not available	Laparoscopy	PC *
Elsner et al. [40]	2016	M	52	Abdominal pain	Single	52 × 45 × 42	Calcified foreign body	Laparoscopy	PC#
Lee et al. [41]	2017	F	61	Abdominal pain	Single	30 (max)	Subserosal myoma	Laparoscopy	PC#
Cheng et al. [3]	2017	M	47	Abdominal pain	Multiple	20–30 (max)	Peritoneal loose body	Laparotomy	PC#
Matsubara et al. [10]	2017	M	70	Urinary frequency	Single	58 (max)	Peritoneal loose body	Laparoscopy	PC#
Huang et al. [4]	2017	M	79	Urinary frequency	Multiple	104 × 83	Not available	Laparoscopy	Left lower quadrant; PC#
						76 × 60			
Obaid and Gehani [42]	2018	M	58	Bilateral flank pain	Single	60 × 48 × 42	Not available	Laparoscopy	PC#
Oom et al. [16]	2018	M	64	Incidental	Single	60 × 60 × 40	Not available	Laparotomy	PC#
Cojocari and David [43]	2018	M	72	Incidental	Single	65 × 58	Not available	Laparotomy	Recto-vesical pouch#
Teklewold et al. [44]	2019	M	50	Abdominal pain	Single	75 × 60 × 50	Not available	Laparotomy	Right paracolic gutter#
Erkan et al. [45]	2019	M	74	Abdominal pain	Single	60 × 50 × 40	Malignant calcific lymph node	Laparotomy	Peritoneal cavity#
Guo et al. [46]	2019	M	49	Abdominal pain	Single	55 × 50	Not available	Laparotomy	PC#
Baert et al. [47]	2019	M	53	Abdominal pain	Single	55 (max)	Foreign object	Laparoscopy	PC#
Dhoot et al. [48]	2020	M	75	Abdominal pain	Single	62 × 58	Latrogenic foreign body	Laparoscopy	PC#
Li et al. [19]	2020	M	46	Incidental	Single	45 × 40 × 33	Teratoma	Laparoscopy	PC#
Allopi et al. [49]	2021	M	79	Abdominal pain	Single	90(max)	Teratoma	Laparotomy	Left upper quadrant#
Ariaya et al. [50]	2021	M	38	Incidental	Single	30 × 30	Not available	Laparotomy	Peritoneal cavity#
Present case		M	69	Abdominal discomfort	Single	30 × 25 × 20	Solitary necrotic nodule	Laparotomy	The second hilar region*

F: female; M: male; PC: pelvic cavity; #: free floating; *: adherence or incarcerated.

## 3 Case report

A 69-year-old man referred to outpatient hepatology because of a 3-month history of abdominal discomfort mainly in the upper quadrants. Other symptoms were denied. The patient’s medical history included hypertension for more than four years. Physical examination findings were normal, and the intra-abdominal mass could not be palpated. The laboratory test results including complete blood count and liver function test results were normal.

**Figure 1:**
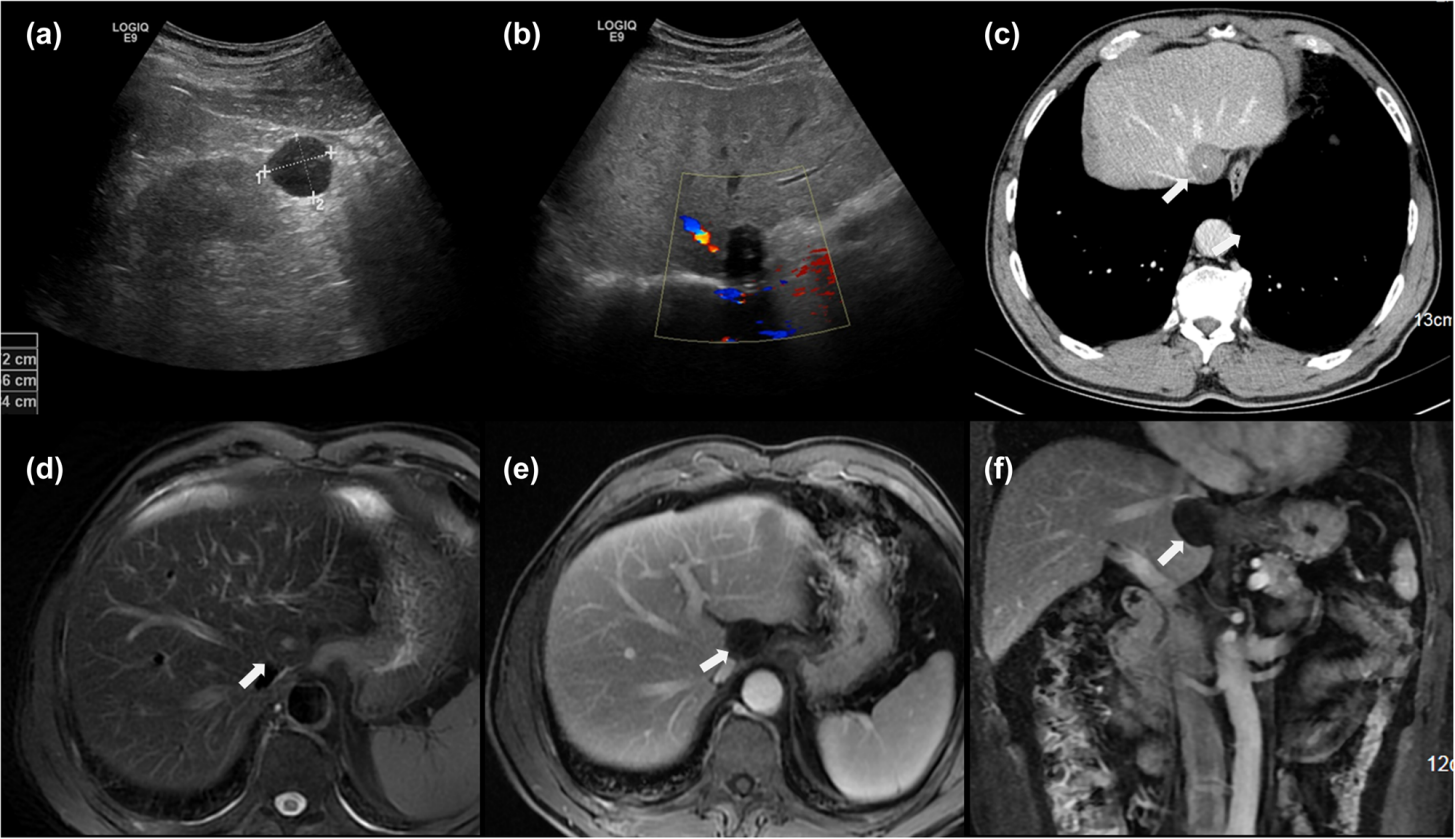
Doppler echocardiogram images (a and b) show a hypoechoic mass at the edge of the hepatic caudate lobe and no obvious blood flow signal in the mass. Contrast-enhanced venous phase CT image of the abdomen (c) reveals a concentric round, well-defined nonenhanced mass (arrow) with central calcification. Fat-suppressed T2-weighted images and gadolinium-enhanced fat-suppressed T1-weighted MR images (d–f) show a hypointense mass as muscle tissue without any enhancement (arrow).

The echocardiography examination revealed a hypoechoic mass with a clear boundary and regular shape in the second hilar region. It contained hyperintense echogenic foci with posterior acoustic shadowing, indicating dense calcifications, and no obvious blood flow signal was detected in the mass (Figure 1a and b). The CT images of the abdomen presented a well-circumscribed, round-like lesion, which was solid with punctate calcification in the center, and no contrast-enhancement was observed in the lesion area by a contrast-enhanced scan (Figure 1c). T1- and T2-weighted MR images of the liver showed a distinct and smoothly marginated hypointense mass near the caudate lobe of the liver, to the same degree as the muscle tissue. Furthermore, no evidence of enhancement was found through the dynamic contrast-enhanced scan (Figure 1d–f).

**Figure 2:**
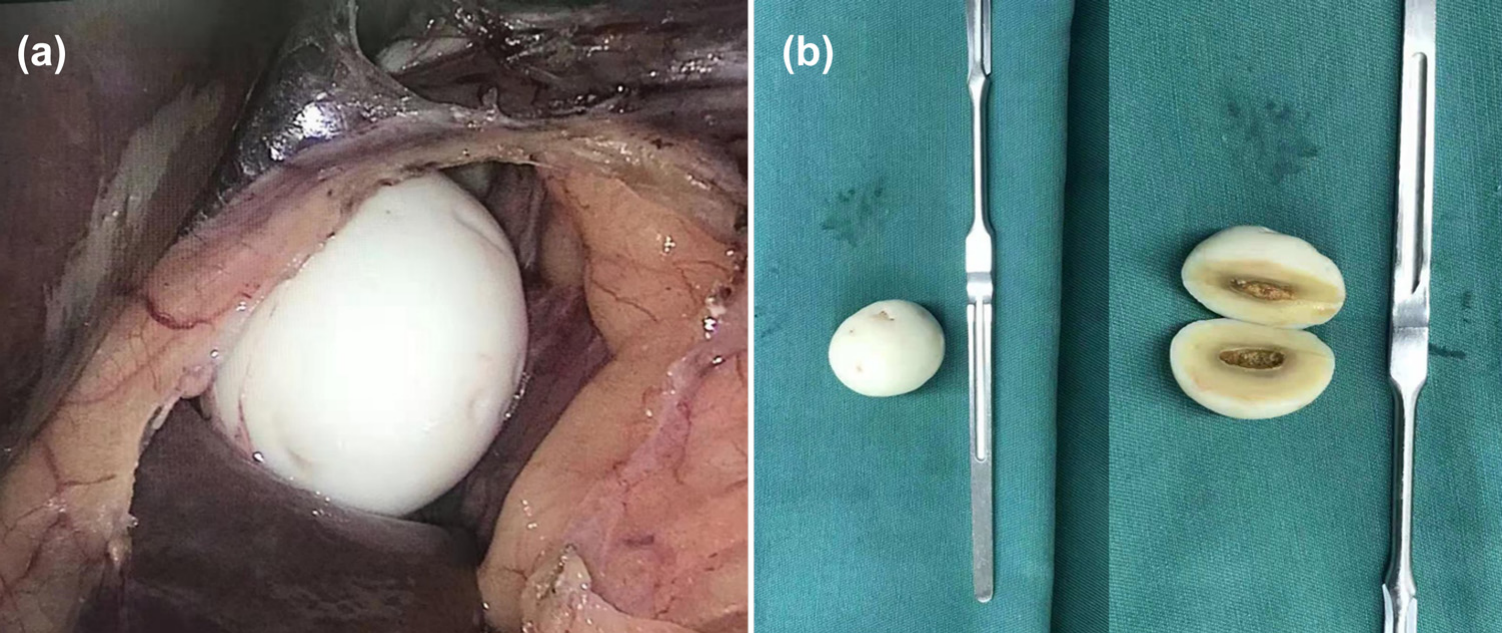
Laparoscopy (a) presents a pure white, elastic, egg-shaped body in the second hilar region and near the caudate lobe. Gross pathologic examination (b) presents a lesion of white in appearance with a smooth surface. The cross-section displays a core filled with yellow material.

**Figure 3:**
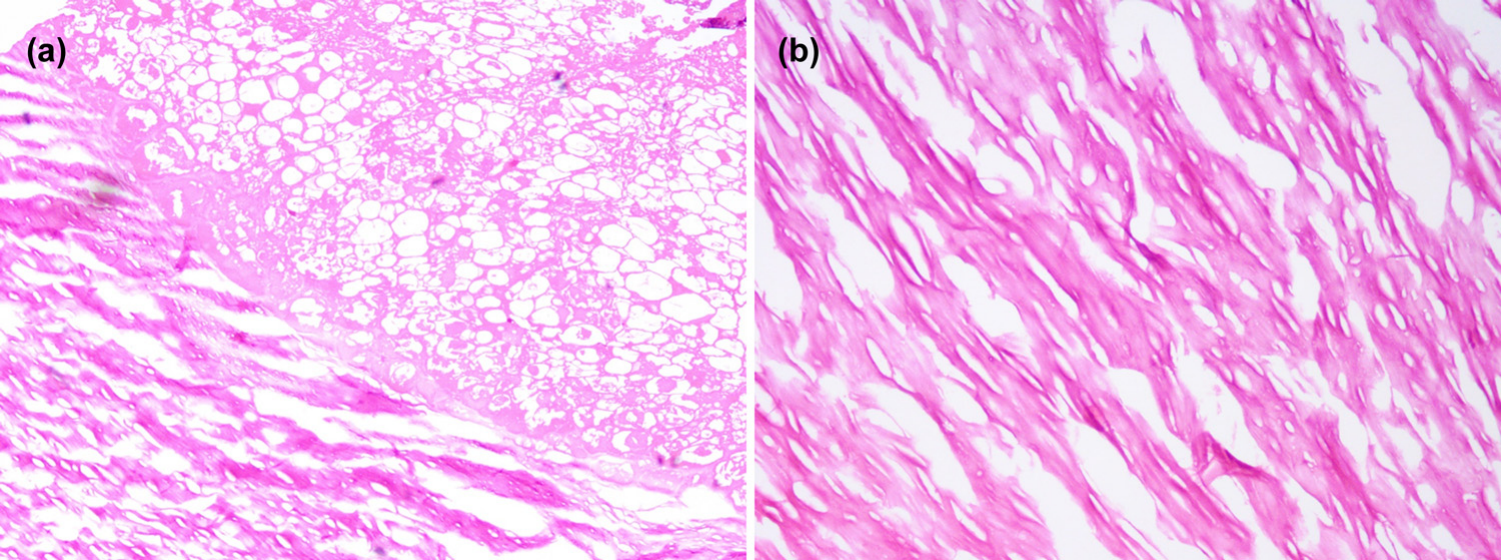
Photomicrograph shows (a) the necrotic and mechanized adipose tissue in the center of the free body, and its periphery surrounded by dense connective tissue and (b) the dense collagen connective tissues around the central degenerated region arranged in concentric circles. (hematoxylin and eosin, a ×40 and b ×100 original magnification).

To confirm the diagnosis and determine the proper treatment strategy, we performed laparoscopic surgery, finding a smooth, free, and “boiled egg-like” mass. This mass was removed by laparoscopy. The PLB was about 3 cm × 2.5 cm × 2 cm in size, elastic, white-color in appearance, and smooth on the surface. The cut surface showed a core with yellow material (Figure 2a and b). Microscopically, the center of the lesion was composed of partially necrotic adipose tissue, and the periphery contained wrapped fibrous tissue with significant hyaline degeneration (Figure 3). The postoperative course was uneventful, with no complications in the perioperative period. The patient was discharged from the hospital six days later.

**Ethics approval and consent to participate:** This study was approved by the Research Ethics Committee of Zhejiang Provincial People’s hospital. Written informed consent was obtained from this patient.

## 4 Discussion

The PLB or “peritoneal mouse,” is an extremely rare clinical entity. Most of the related reports are based on individual cases. As shown in Table 1, a literature search found 49 cases of PLBs. Interestingly, most PLBs occurred in males over age 50, the age span of patients at the time of diagnosis ranges from 2 months to 79 years, and the incidence rate ratio between males and females is 42:8. PLBs are usually greater than 2 cm in size and most cases are solitary, multiple lesions (≥2) in a case are rare (4 out of 50 cases, 8%) [1,2,3,4]. Small PLBs rarely cause symptoms, and it is mostly found accidentally; however, larger PLBs can cause symptoms by compressing surrounding structures or internal organs. Of 50 PLBs, 36 (72%) were symptomatic, the symptoms described in the literature range from abdominal discomfort, pain and intermittent constipation [5], intestinal obstruction [2,6,7] to bladder irritation [4,8,9,10], with the most common symptom being abdominal pain or discomfort (19 out of 50 cases, 38%).

Though the exact pathogenesis of PLB is unknown, the tissue in the center of the PLB is the prerequisite for the formation of the free body. At present, scholars speculated that the tissue may come from the following substances [11]: (1) the fat sags of the intestine; (2) the fat in the greater omentum; (3) the lymph nodes of fat deposition; (4) the fat in the pancreas; and (5) autoamputated adnexa, other origins of PLBs can also be uterine fibroids, ovarian masses, foreign bodies, etc., [7,12,13,14]. The most frequently described etiology is the chronic torsion of an epiploic appendix [11,15], which undergoes a sequential process [16] to form PLBs: (1) torsion of the epiblastic appendix; (2) ischemia; (3) saponification; (4) calcification; and (5) separation of the colon due to atrophy of the pedicle. The pronucleus can further undergo saponification and calcification, which will stimulate the peritoneal cavity to produce exudate. The protein-rich exudate continuously accumulates and wraps around the pronucleus. Moreover, due to the higher temperature in the peritoneal cavity, the coating layer is denatured and hence, causes lamellar hardening. Most often, such a free body remains in the peritoneal cavity for a long time. As time changes, its volume gradually increases, finally forming a “boiled egg-like” structure [5,6].

PLB is free in the peritoneal cavity and has no specific position, it will move with the change in body position or respiratory and gastrointestinal movement, but the migration may be substantially impeded by the adhesions or abdominal organs in the vicinity [6,9,17,18]. PLBs are usually found in the pelvis cavity because they tend to gravitate to the most dependent part of the peritoneal cavity. Because the condition is rare, most physicians and radiologists have insufficient knowledge in this regard. A good understanding of its specific imaging features before surgery can help prevent misdiagnosis and avoid unnecessary surgical exploration. PLBs typically appear as a central calcified nodule on CT [8,19] and does not exhibit obvious contrast enhancement. MRI showed a smooth-surfaced, egg-shaped lesion, which was seen as a low-intensity mass on both T1- and T2-weighted MRI, to the same degree as the muscle tissue [20]. Under ultrasound, it showed a hypoechoic mass with a clear boundary and regular shape as well as irregular hyperechoic in the center, there was no clear blood flow signal inside the mass. Although the mass is easy to change location under probe compression, we had not seen this manifestation during ultrasound examination, and no significant change in PLB position was observed in the images of the multiple examination mode in this case. A benign hepatic tumor or tumor-like lesion was preliminarily diagnosed, such as solitary necrotic nodule. However, the mass was found free when the ultrasonic scalpel was used to free the second hepatic portal and left hepatic vein during the operation. We hence speculate that the PLB sometimes gets stuck in some narrow space or crypt, thus losing its moving characteristics. This can explain the patient’s upper abdominal discomfort in the past three months, and also increase the difficulty of preoperative diagnosis. No similar situation has been reported in the literature before. The microscopic appearance of this case is consistent with the one described in the literature that the typical pathological feature of PLB is fat necrosis and calcification in the center part, and acidophilic concentric round fibrous structure in the periphery [20], and the pathologic features of acellular fibrous nodule with central necrotic fatty tissue and without any epithelium or muscle components can make a final diagnosis. The differential diagnosis of PLBs includes dermoid cyst, teratoma, stromal tumor, calcified hysteromyoma, ectopic pregnancy, and tuberculous granuloma [1,11]. US, CT, and MRI can be performed to distinguish PLBs from other pathologies before operation.

## 5 Conclusion

Notably, small asymptomatic PLBs often require no special treatment and can be monitored by regular medical reviews. Nonetheless, surgery is the main treatment in symptomatic patients, which can not only relieve symptoms but also promote definitive diagnosis. Exploratory laparoscopy is a recommended means for the detection and management of PLBs, which reduces surgical damage, minimizes postoperative complications, and shortens recovery time [11].
